# Low Temperature Fabrication for High Performance Flexible CsPbI_2_Br Perovskite Solar Cells

**DOI:** 10.1002/advs.201801117

**Published:** 2018-09-15

**Authors:** Hong Jiang, Jiangshan Feng, Huan Zhao, Guijun Li, Guannan Yin, Yu Han, Feng Yan, Zhike Liu, Shengzhong (Frank) Liu

**Affiliations:** ^1^ Key Laboratory of Applied Surface and Colloid Chemistry Ministry of Education Shaanxi Key Laboratory for Advanced Energy Devices Shaanxi Engineering Lab for Advanced Energy Technology School of Materials Science and Engineering Shaanxi Normal University Xi'an 710119 China; ^2^ Department of Applied Physics The Hong Kong Polytechnic University Hung Hom Kowloon 999077 Hong Kong; ^3^ Dalian National Laboratory for Clean Energy iChEM Dalian Institute of Chemical Physics Chinese Academy of Sciences Dalian 116023 China

**Keywords:** CsPbI_2_Br, flexible, high performance, high stability, low temperature

## Abstract

All‐inorganic CsPbX_3_‐based perovskites, such as CsPbI_2_Br, show much better thermal and illumination stability than their organic–inorganic hybrid counterparts. However, fabrication of high‐quality CsPbI_2_Br perovskite film normally requires annealing at a high temperature (>250 °C) that is not compatible with the plastic substrate. In this work, a Lewis base adduct‐promoted growth process that makes it possible to fabricate high quality CsPbI_2_Br perovskite films at low temperature is promoted. The mechanism is attributed to synthesized dimethyl sulfoxide (DMSO) adducts which allow a low activation energy route to form CsPbI_2_Br perovskite films during the thermal annealing treatment. A power conversion efficiency (PCE) of 13.54% is achieved. As far as it is known, this is the highest efficiency for the CsPbI_2_Br solar cells fabricated at low temperature (120 °C). In addition, the method enables fabrication of flexible CsPbI_2_Br PSCs with PCE as high as 11.73%. Surprisingly, the bare devices without any encapsulation maintain 70% of their original PCEs after being stored in ambient air for 700 h. This work provides an approach for preparing other high performance CsPbX_3_‐based perovskite solar cells (PSCs) at low temperature, particularly for flexible ones.

Recently, all‐inorganic cesium lead halide perovskites (CsPbX_3_, X = I, Br, Cl, or mixed halides) have drawn extensive research efforts owing to their superior thermal and illumination stability compared to their organic–inorganic hybrid counterparts.^1^, ^2^, ^3^, ^4^ The CsPbX_3_‐based inorganic perovskites have been demonstrated to be another promising and novel candidates for photovoltaic applications.^5^, ^6^ However, most reported high performance CsPbX_3_ perovskite solar cells (PSCs) need to be prepared at high temperature (>250 °C) to overcome the crystallization energy barrier for the black cubic perovskite phase(α‐phase).^7^, ^8^, ^9^ Such high heating temperature will lead to a high fabrication cost and process complexity. Moreover, high temperature fabrication will limit the selection of substrates in the device and the possibility of application inflexible devices. When the CsPbX_3_ perovskites are prepared at low temperature (≤150 °C), they generally exhibit a yellow orthorhombic phase (δ‐phase) and poor crystallinity that are unsuitable for solar cell applications.^10^ Up to now, it is still challenging to fabricate pure α‐phase CsPbX_3_ perovskite films with high quality at low temperature, owing to a trade‐off between processing temperature and device performance. Therefore, it is highly desirable to develop low temperature processed methods for preparing high quality CsPbX_3_ inorganic perovskite films which can not only simplify the complicated device process, but also promote emerging flexible device technologies.

Among CsPbX_3_‐based perovskites, CsPbI_2_Br is assumed to be a promising absorber layer for its balance of the trade‐off between the bandgap and phase stability. In this scenario, recently, the CsPbI_2_Br PSCs have been developed rapidly, which not only gave rise to a record power conversion efficiency (PCE) over 14%, but also exhibited good thermal and illuminated stability.^11^ However, it is still a serious challenge to synthesize pure α‐phase CsPbI_2_Br with superior photovoltaic performance at low temperature. Until now, there have been few reports on the preparation of CsPbI_2_Br PSCs at low temperature. For example, Wang et al. applied HPbI_3_ to replace PbI_2_ as a precursor for preparing CsPbI_2_Br film.^12^ It has been found that the precursor solution (CsI+PbBr_2_+HPbI_3_) can decrease the formation energy barrier for the α‐CsPbI_2_Br phase to help form a compact and pinhole‐free film. The champion CsPbI_2_Br PSC achieves a PCE of 10.56% and exhibits long‐term phase stability at 130 °C. Lau et al. incorporated strontium into CsPbI_2_Br to prepare PSCs at low temperature (100 °C).^13^ The champion CsPb_0.98_Sr_0.02_I_2_Br PSCs delivered a highest PCE of 11.3% and better thermal stability. Most recently, Rao et al. controlled the morphology of CsPbI_2_Br film by dimethyl sulfoxide (DMSO) solvent and obtain a pure α‐phase CsPbI_2_Br at low temperature (120 °C).^14^ The maximum efficiencies of the optimized rigid and flexible devices are only 10.4% and 7.3%, respectively. Therefore, up to now, the PCEs of rigid and flexible CsPbI_2_Br PSCs prepared at low temperature (≤150 °C) are still lower than 12% and 8%, respectively.

In this work, we propose a low temperature scheme of DMSO‐adduct promoted process (DAPP) for high quality perovskite films. The PbI_2_(DMSO), PbBr_2_(DMSO) adducts are obtained via treating lead halides with DMSO under low temperature conditions (60 °C), which can efficiently prevent rapid reaction of precursors and slow down the crystal growth. On the other hand, DMSO adducts can also reduce the formation energy of CsPbI_2_Br perovskite that needs to be overcome by conventional precursors (PbI_2_ and PbBr_2_). By using the as‐prepared DMSO adducts instead of commercial PbI_2_ and PbBr_2_ for the perovskite precursor solution, a high‐quality perovskite film can be successfully prepared under low temperature conditions (120 °C), which is free of pinholes and impurities, and has high crystallinity and stability. Using the low temperature crystallized perovskite films, a high performance CsPbI_2_Br PSC with PCE of 13.54% is fabricated (the current best CsPbI_2_Br PSCs fabricated at low temperature (≤150 °C) have a PCE of <11%).^12^ In addition, the low temperature DAPP method enables the fabrication of flexible CsPbI_2_Br PSCs with a high PCE of 11.73%. Furthermore, the flexible CsPbI_2_Br PSCs are very stable, which can maintain 90% and 70% of their original PCEs after being bent under a curvature radius of 5 mm and stored in an ambient environment for 700 h, respectively. This work not only provides a novel route for preparing high‐quality CsPbI_2_Br perovskites at low temperature but also represents an important step for their application in high‐performance and low‐cost flexible electronics.

In thermodynamics, activation energy or formation energy represents the energy required for a chemical reaction. According to the recent literature, the reaction coordinate diagram of the all‐inorganic perovskite material formation via different pathways is presented in **Figure** **1**.^15^, ^16^, ^17^ In the conventional pathway based on pure PbI_2_ and PbBr_2_, the formation of all‐inorganic perovskite materials needs to overcome a large energy barrier (*E*_a_). Interestingly, it is found that the formation of all‐inorganic perovskite materials undergoes two‐stage reactions in a Lewis base adduct‐promoted pathway, PbI_2_(Lewis base)*_X_* formation and perovskite formation. The first step needs to overcome a small energy barrier (*E*_a1_) from PbI_2_ to PbI_2_(Lewis base)*_X_*, meaning that PbI_2_(Lewis base)*_X_* can be obtained at low temperature for a short time. *E*_a2_ is the Lewis base adduct‐promoted pathway from PbI_2_(Lewis base)*_X_* to perovskite, which is substantially lower than that of the conventional pathway (*E*_a_). Therefore, a high‐quality perovskite film can be readily obtained at low temperature for a short time through the Lewis base adduct‐promoted growth.

**Figure 1:**
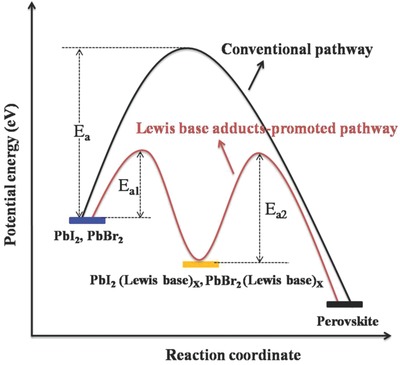
The reaction coordinate diagram of the inorganic perovskite formation via the conventional pathway and DSMO‐adduct promoted pathway. *E*_a_, *E*_a1_, and *E*_a2_ are the activation energies for the reactions.

Therefore, in order to obtain high quality inorganic perovskite materials at low temperature, in this work, we propose a DMSO adduct‐promoted process (DAPP) for formation of all‐inorganic perovskite CsPbI_2_Br. The DAPP is a two‐stage reaction pathway with a lower activation energy and different from the conventional single pathway with a higher activation energy. First, the DMSO adducts, PbI_2_(DMSO), and PbBr_2_(DMSO), are synthesized at 60 °C as precursors to replace PbI_2_ and PbBr_2_ in conventional all‐inorganic perovskite solution. On the one hand, the DMSO adducts can effectively retard the fast reaction between PbI_2_, PbBr_2_, and CsI during the evaporation of solvent. On the other hand, the DMSO adducts can reduce the formation energy of CsPbI_2_Br perovskite as discussed before.

The fabrication of PbI_2_(DMSO) and PbBr_2_(DMSO) adducts contains two steps, namely, I) adding toluene as a nonsolvent into PbI_2_ or PbBr_2_ solution dissolved in DMSO to obtain white precipitation and II) annealing the white precipitation at 60 °C for 24 h.^4^ The formation of DMSO adducts is confirmed by X‐ray powder diffraction (XRD) (Figure S1a,b, Supporting Information) and ultraviolet–visible (UV–Vis) absorption spectra (Figures S2a,b, Supporting Information). The XRD patterns of the white precipitations are well matched with PbI_2_(DMSO) and PbBr_2_(DMSO) phase reported in literature.^18^ Compared with PbI_2_ powders, the absorption edge of PbI_2_(DMSO) precipitation is removed to short wavelength corresponding to the change from yellow‐colored PbI_2_ powered to colorless PbI_2_(DMSO) adducts, which is in agreement with the reported results.^18^

Then the CsPbI_2_Br precursor films are deposited on the rigid substrate by coating precursor solution via antisolvent ways.^19^, ^20^ To evaluate the influence of the DAPP on the crystallization process of CsPbI_2_Br perovskite, the CsPbI_2_Br precursor films without annealing are stored in a glove box for 24 h (Figure S3, Supporting Information). It is found that the CsPbI_2_Br precursor film without DAPP turns black, indicating crystallization of CsPbI_2_Br film. Conversely, the CsPbI_2_Br precursor film with DAPP shows dark brown, meaning that the DAPP can slow down the crystallization process of CsPbI_2_Br. In order to further confirm the influence of the DAPP, the CsPbI_2_Br precursor films are annealed at low temperature (35 °C) for different times (Figure S4, Supporting Information), the CsPbI_2_Br films without and with DAPP turn black and light brown after being annealed at 35 °C for 35 min, respectively. Therefore, it is concluded that the DAPP can control the crystallization kinetic of CsPbI_2_Br perovskite, which is crucial for high quality CsPbI_2_Br film.^21^

**Figure** **2**a shows XRD patterns of CsPbI_2_Br perovskite films with and without DAPP annealed at 65 °C or 120 °C for 10 min on a hotplate. Many studies have found that the cubic α‐CsPbI_2_Br phase is often obtained when the annealing temperature is higher than 250 °C.^22^, ^23^, ^24^ In our work, it is interesting to find that a pure cubic α‐CsPbI_2_Br phase transition easily occurs by using DMSO adducts as precursor even at 65 °C for 10 min. The characteristic Bragg peaks of CsPbI_2_Br thin films at 2θ = 14.6°, 29.5° show that both films are well‐oriented in the cubic (100) direction. The peak splitting of the (100) and (200) planes is observed in the control film (Figure S5, Supporting Information) annealed at 65 °C for 10 min, indicating the separate growth of I‐rich (2θ = 14.4°, 29.1°) crystals.^25^ The XRD peak intensity of CsPbI_2_Br films is enhanced after annealing at high temperature (120 °C), which benefits from the improved crystallinity of the CsPbI_2_Br film. The intensity ratio of CsPbI_2_Br films with and without DAPP is 1.2, which means that the quality of CsPbI_2_Br films is improved after DAPP optimizing.

**Figure 2:**
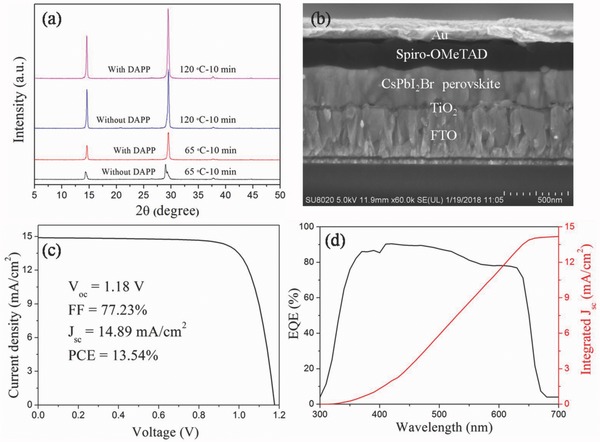
a) XRD patterns of CsPbI_2_Br films without and with DAPP annealed at 65 or 120 °C for 10 min. b) The cross‐sectional view of the completed CsPbI_2_Br PSCs with DAPP. c) *J*–*V* curve of the champion CsPbI_2_Br PSC under one sun (AM 1.5G illumination). d) The corresponding external quantum efficiency (EQE) spectrum together with the integrated *J*_SC_ of the champion CsPbI_2_Br PSC.

The UV–Vis absorption spectra of CsPbI_2_Br films with and without DAPP are shown in Figure S6 (Supporting Information). The CsPbI_2_Br film with DAPP exhibits an increased absorption over the entire absorption range compared to CsPbI_2_Br film without DAPP, which is due to its higher crystallinity (Figure 2a). To investigate the influence of DAPP on photogenerated charge carriers in the CsPbI_2_Br films, the steady‐state photoluminescence (PL) and time‐resolved PL (TRPL) measurements are performed (Figure S7, Supporting Information). The PL intensity of the CsPbI_2_Br film with DAPP is much higher than that of the film without DAPP, suggesting a significantly reduced nonradiative recombination rate within the perovskite film. The PL decay lifetimes of the CsPbI_2_Br films are determined by TRPL measurements (Figure S7b and Table S2, Supporting Information). The CsPbI_2_Br film with DAPP shows a much longer lifetime (11.57 ns) than that of the film without DAPP (3.41 ns), indicating that the improved crystalline quality of CsPbI_2_Br film can significantly prolong the lifetimes of carriers.^26^

Figure 2b shows the cross‐sectional scanning electron microscopy (SEM) image of the completed CsPbI_2_Br PSC on the rigid substrate. The device has a structure of (FTO/TiO_2_/CsPbI_2_Br/Spiro‐OMeTAD/Au). The energy band levels of the CsPbI_2_Br PSC are shown in Figure S8 (Supporting Information). In this device, the 300 nm CsPbI_2_Br perovskite layer is prepared by PbI_2_(DMSO), PbBr_2_(DMSO) and CsI, which is free of pinholes and has a compact surface (Figure S9a, Supporting Information) that is critical for the photovoltaic performance of CsPbI_2_Br PSC. The DMSO in DMSO adducts, PbI_2_(DMSO) and PbBr_2_(DMSO), could not easily escape from the precursor film after spin‐coating, which could enhance the mass transport and diffusion, slow down the rate of crystallization, and eventually improve the film quality.^26^ In contrast, when the control solution without DMSO adducts is deposited onto the TiO_2_ layer, some pinholes can be observed on the film probably due to the rapid crystallization (Figure S9b, Supporting Information), which could lead to the lower PCEs and instability for inorganic PSCs. This result indicates that high quality CsPbI_2_Br perovskite film can be obtained by using DMSO adducts through a low temperature solution process. The representative current density–voltage (*J*–*V*) curve of the CsPbI_2_Br PSCs is given in Figure 2c. The champion device achieved a *V*_OC_ of 1.18 V, a FF of 77.23%, a *J*_SC_ of 14.89 mA cm^−2^, and a PCE as high as 13.54%, which is one of the highest PCEs among the all‐inorganic perovskite PSCs to date and much higher than the PCEs of the reported all‐inorganic perovskite PSCs that prepared at low temperature (**Table** **1**).^27^, ^28^, ^29^ The device also shows a negligible *J*–*V* hysteresis (Figure S10, Supporting Information). Figure 2d shows the corresponding external quantum efficiency (EQE) of the CsPbI_2_Br device with an integrated photocurrent density of 14.20 mA cm^−2^, which is close to the *J*_SC_ derived from the *J*–*V* measurement.

**Table 1:** Summary of the all‐reported CsPbI_2_Br PSCs prepared at low temperature

Configuration	Temperature [°C]	*V*_OC_ [V]	*J*_SC_ [mA cm^−2^]	FF [%]	PCE [%]	Ref.
FTO/TiO_2_/CsPbI_2_Br/spiro‐ OMeTAD/Au (rigid)	130	1.13	13.6	68.6	10.6	12
FTO/TiO_2_/CsPbI_2_Br/P3HT/Au (rigid)	100	0.96	13.4	59.8	7.7	13
FTO/TiO_2_/CsPb_0.95_Sr_0.05_I_2_Br/P3HT/Au (rigid)	100	1.06	14.9	70.9	11.3	13
ITO/NiO_x_/CsPbI_2_Br/C_60_/Bathocuproine (BCP)/Ag (rigid)	120	1.05	12.6	78.7	10.4	14
PET/ITO/NiO_x_/CsPbI_2_Br/C_60_/ BCP/Ag (flexible)	120	0.97	11.5	65.0	7.3	14
FTO/TiO_2_/CsPbI_2_Br/spiro‐ OMeTAD/Au (rigid)	120	1.18	14.9	77.2	13.5	This work
PET/ITO/Nb_5_O_2_/CsPbI_2_Br/spiro‐OMeTAD/Au (flexible)	130	1.19	14.6	67.3	11.7	This work

Considering that the high performance CsPbI_2_Br PSCs have been successfully fabricated on rigid substrate under low temperature (120 °C), the high performance flexible CsPbI_2_Br PSCs on flexible substrate are expected. The flexible CsPbI_2_Br PSCs are further fabricated by utilizing ITO/polyethylene terephthalate (PET) as the conductive transparent electrode/substrate. Nb_2_O_5_ film is e‐beam evaporated at room temperature as an electron transport layer.^30^ The SEM image of Nb_2_O_5_ film on flexible PET substrate is shown in **Figure** **3**a. The Nb_2_O_5_ film has a thickness of 60 nm and shows a smooth surface morphology with uniform grain structure. As shown in Figures 3b,c, the CsPbI_2_Br film on flexible substrate with DAPP has a dense and smooth surface, and passivated grain boundary. In contrast, the control CsPbI_2_Br film without DAPP has a rough surface and some cracks, which is probably due to the rapid crystallization process.^25^, ^31^

**Figure 3:**
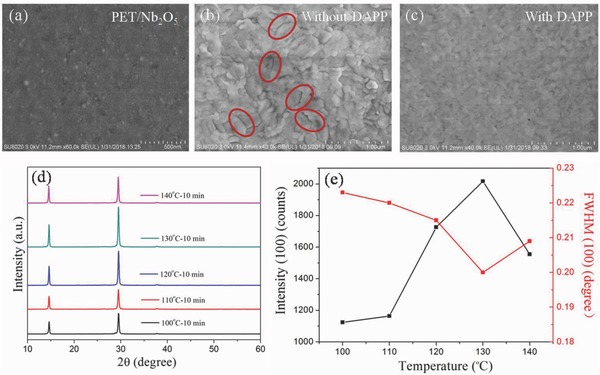
a) SEM image of Nb_2_O_5_ film coated on flexible PET substrate. SEM images of the CsPbI_2_Br perovskite films coated on PET/ITO/Nb_2_O_5_ substrate b) without and c) with DAPP. d) XRD patterns and e) the diffraction intensity and full width at half maxima (FWHM) of (100) peaks of CsPbI_2_Br perovskite films with DAPP annealed at different temperatures.

In order to investigate the effect of temperature on the crystallization of CsPbI_2_Br films on the flexible substrates, the CsPbI_2_Br solution with DMSO adducts are spin‐coated on Nb_2_O_5_/ITO/PET substrate and then annealed at different temperatures (100, 110, 120, 130, 140 °C) on a hotplate to form a 300 nm CsPbI_2_Br perovskite film. The XRD results of CsPbI_2_Br films are shown in Figure 3d,e, the pure CsPbI_2_Br thin films show the Bragg peaks at 14.6° and 29.5°. As the annealing temperature increases, the intensities of the diffraction (100) peaks increase while the full width at half maxima (FWHM) decreases due to the increased crystallinity of perovskite film. However, with further increases of the temperature to 140 °C, the intensity of the diffraction peak decreases while the FWHM increases, which is probably due to the influence of thermal deformation behavior of PET substrates.^32^ The effect of different annealing times on the crystallization of CsPbI_2_Br films at the optimal annealing temperature is also investigated (Figure S11, Supporting Information). The intensities of (100) peak increase from 2 to 30 min due to the increased crystallinity of perovskite film. With further annealing up to 40 min, the change of the (100) peak intensity is not obvious.

To build up flexible CsPbI_2_Br PSCs, as shown in **Figure** **4**a, a conventional n–i–p architecture (PET/ITO/Nb_2_O_5_/CsPbI_2_Br/Spiro‐OMeTAD/Au) is adopted. The energy band levels of the flexible CsPbI_2_Br PSC are shown in Figure S12 (Supporting Information). The effect of different annealing temperatures on the photovoltaic performance of the CsPbI_2_Br PSCs is investigated. The detailed photovoltaic parameters are summarized in **Table** **2**. The statistical distributions of the photovoltaic parameters based on 40 individual planar PSCs are summarized in Figure S13 (Supporting Information). In short, the photovoltaic parameters of the CsPbI_2_Br PSCs are increased with increasing temperature due to the increased crystallinity of perovskite film, and the CsPbI_2_Br PSC exhibits the best performance when the temperature is 130 °C. When annealing temperature up to 140 °C, the performance of device decreases, which may be due to the influence of thermal deformation behavior of PET substrates.^32^, ^33^ Meanwhile, the effects of annealing times (2, 10, 20, and 40 min) at 130 °C on the performance of the CsPbI_2_Br PSCs are also investigated. The detailed photovoltaic parameters and their statistical distributions are summarized in Table S1 and Figure S14 (Supporting Information), respectively. The optimized annealing time at 130 °C is 10 min. The *J*–*V* curve of the champion flexible PSC annealed at optimized condition (130 °C‐10 min) is shown in Figure 4b. The PCE of the flexible CsPbI_2_Br PSC can reach up to 11.73% with a *V*_OC_ of 1.19 V, a FF of 67.25%, and a *J*_SC_ of 14.61 mA cm^−2^. In contrast, the control flexible CsPbI_2_Br PSC without DAPP only exhibits a PCE of 9.48% with a *V*_OC_ of 1.08 V, a FF of 65.0% and a *J*_SC_ of 13.55 mA cm^−2^ (Figure S15, Supporting Information). Obviously, all the photovoltaic parameters of CsPbI_2_Br PSCs with DAPP have higher values than the control samples. Compared with the control device, the PCE of CsPbI_2_Br PSCs with DAPP is increased about 24%. Figure 4c shows the corresponding EQE of the optimized flexible CsPbI_2_Br device with an integrated *J*_SC_ value of 14.28 mA cm^−2^, which agrees well with the *J*–*V* measurements. To confirm the reliability of the *J*–*V* measurements, the current density and PCE at the maximum power point (0.94 V) are recorded as functions of time, as presented in Figure 4d. When measured for a period of light soaking times, a stabilized efficiency of 11.63% with a stable *J*_SC_ of 12.37 mA cm^−2^ is obtained, which is very close to the efficiency value obtained by the *J*–*V* measurements.

**Figure 4:**
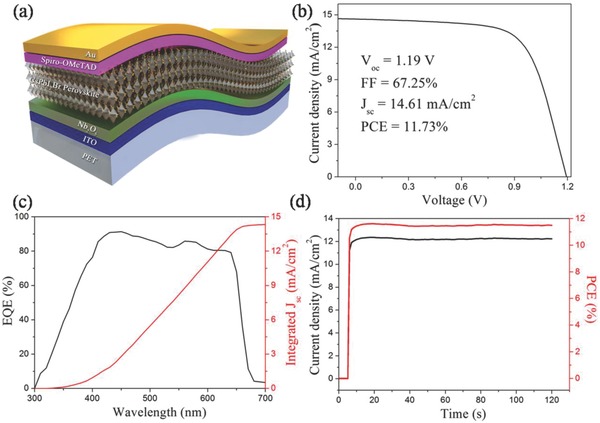
a) Schematic diagram of flexible CsPbI_2_Br PSC with a structure of PET/ITO/Nb_2_O_5_/CsPbI_2_Br/spiro‐OMeTAD/Au. b) *J*–*V* curve of the champion flexible CsPbI_2_Br PSC with DAPP under 1 sun (AM 1.5G illumination). c) The EQE spectrum of flexible PSC based on CsPbI_2_Br film with DAPP. d) Stabilized short‐circuit photocurrent density and efficiency of flexible CsPbI_2_Br PSC with DAPP.

**Table 2:** Comparison of the performance parameters of the CsPbI_2_Br PSCs with DAPP annealed at different temperatures

Conditions	*V*_OC_ [V]	*J*_SC_ [mA cm^−2^]	FF [%]	PCE [%]
100 °C‐10 min	1.16	14.33	65.71	10.91
110 °C‐10 min	1.17	14.39	65.79	11.03
120 °C‐10 min	1.17	14.47	67.37	11.40
130 °C‐10 min	1.19	14.61	67.25	11.73
140 °C‐10 min	1.12	14.17	66.34	10.51

To demonstrate the flexible property of CsPbI_2_Br PSCs, the mechanical bending tests of flexible CsPbI_2_Br PSCs are carried out with different curvature radii. The *J*–*V* curves of the flexible devices before and after bending at curvature radii of 12, 7, and 5 mm for 300 cycles are shown in **Figures** **5**a–c, respectively. A vernier caliper is applied to define the curvature radius.^34^, ^35^, ^36^ The performance of the flexible CsPbI_2_Br PSC exhibits no significant degradation after bending at a curvature radius of 12 mm for 300 cycles. In addition, the flexible CsPbI_2_Br PSC retained more than 90% of its initial PCE value even after bending at a curvature radius of 5 mm for 300 cycles, proving excellent mechanical stability.

**Figure 5:**
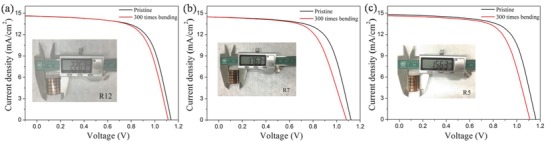
*J*–*V* curves of the flexible CsPbI_2_Br PSCs before and after 300 bending cycles with the curvature radius of a) 12 mm, b) 7 mm, and c) 5 mm, respectively. The insets show the corresponding bending images.

To examine the effect of the DAPP method upon the device air stability, CsPbI_2_Br films and PSCs are stored without encapsulation at room temperature in ambient air at ≈30% relative humidity (RH). As shown in **Figure** **6**a,b, CsPbI_2_Br films annealed at 65 °C with and without DAPP undergo a complete degradation after storing in air for 4 h. When the annealing temperature is increased to 120 °C, CsPbI_2_Br film without DAPP shows a significant degradation, whereas the CsPbI_2_Br film with DAPP remains in its initial state, proving that DAPP method can enhance the stability of CsPbI_2_Br film that annealed at low temperature. Figure 6c,d shows the long‐term air stability of flexible CsPbI_2_Br PSCs with or without DAPP, the flexible PSCs with DAPP still retain ≈70% of the initial efficiencies after 700 h aging in air. Such a long‐term stability of flexible CsPbI_2_Br PSCs is closely related to the high‐quality of CsPbI_2_Br film. In contrast, the efficiencies of the control device without DAPP rapidly decrease to less than 60% of their initial values within 5 h.

**Figure 6:**
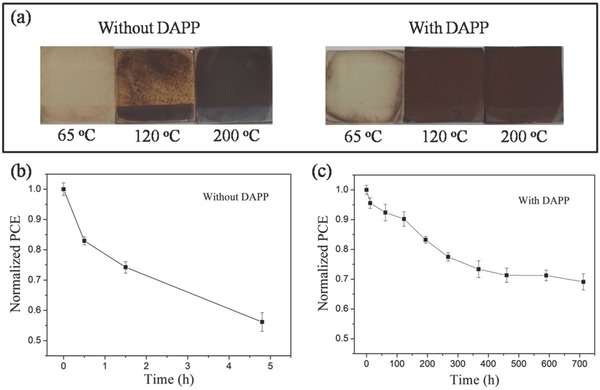
a) Comparison of the air stability (humidity: ≈30%) of the CsPbI_2_Br perovskite films without and with DAPP annealed at different temperatures. The air stability (humidity: ≈30%) of the flexible CsPbI_2_Br PSCs b) without and c) with DAPP for different time periods.

Meanwhile, the thermal (100 °C) or high‐humidity (RH 55%, 25 °C) stabilities of flexible CsPbI_2_Br PSCs with or without DAPP are also investigated, respectively (Figure S16, Supporting Information). It turns out that the CsPbI_2_Br PSCs with DAPP have better thermal and humidity stabilities than their counterparts without DAPP. However, the stability performance of CsPbI_2_Br PSCs is still inferior to the CsPbBr_3_ and CsPbIBr_2_ PSCs with the lower iodide content.^37^, ^38^ Further enhancements in stability can be expected via reducing the content of iodide and/or replacing moisture sensitive Spiro‐OMeTAD with dopant‐free hole transporting materials.

In summary, we have demonstrated a low temperature DAPP method to fabricate high quality CsPbI_2_Br film through reducing the reaction energy of precursors by using PbI_2_(DMSO) and PbBr_2_(DMSO) to replace PbI_2_ and PbBr_2_. This method can obtain a high quality and stable CsPbI_2_Br film even at 120 °C. A CsPbI_2_Br PSC with PCE of 13.54% is fabricated on rigid substrate, showing negligible hysteresis. In addition, the low temperature DAPP method based on DMSO adducts enables the fabrication of the flexible CsPbI_2_Br PSCs with a highest reported PCE of 11.73%, the flexible CsPbI_2_Br PSCs can maintain 90% and 70% of their original PCE after being bent under a curvature radius of 5 mm and stored in an ambient environment for 700 h, respectively. Therefore, this work not only proves the feasibility of producing highly efficient CsPbI_2_Br PSCs on rigid substrates at low temperature, but also opens a new avenue to realize the high performance flexible CsPbI_2_Br PSCs on flexible substrates.

## Experimental Section

*Materials*: The PbI_2_(DMSO), PbBr_2_(DMSO) adducts were prepared by dissolving the appropriate amount of the commercial PbI_2_ (99.99%, Alfar Aesar) and PbBr_2_ (99.99%, Alfar Aesar) in DMSO with heating (60 °C) and stirring, respectively. Then 40 mL of antisolvent (toluene, acetone or isopropanol) was slowly added into the PbI_2_ or PbBr_2_ solution to precipitate the DMSO adducts. After the precipitation was completely produced, it was filtered and dried in a vacuum oven for 24 h to get white PbI_2_(DMSO)_2_ and PbBr_2_(DMSO)_2_ powders. The PbI_2_(DMSO) and PbBr_2_(DMSO) adducts were obtained by annealing PbI_2_(DMSO)_2_ and PbBr_2_(DMSO)_2_ in a vacuum oven at 60 °C for 24 h, respectively.

*Device Fabrication*: The rigid all‐inorganic CsPbI_2_Br PSCs were fabricated with a structure of FTO/TiO_2_/CsPbI_2_Br/spiro‐OMeTAD/Au. FTO/glass substrates were successively cleaned in an ultrasonic bath of water, acetone and isopropanol alcohol for 10 min. Then the surface of substrates was treated under UVO for 15 min to make a hydrophilic surface. TiO_2_ layers (40 nm) were prepared by a chemical bath deposition method as electron transport layers. CsPbI_2_Br perovskite layers were prepared by spin coating the perovskite precursors (0.208 g PbI_2_, 0.165 g PbBr_2_, 0.234 g CsI, or 0.243 g PbI_2_(DMSO), 0.200 g PbBr_2_(DMSO), 0.234 g CsI) in mixed solvent of *N*,*N*‐dimethylformamide (DMF) and DMSO (4:1, v/v) with a concentration of 0.9 m at 1000 rpm for 10 s and 4000 rpm for 40 s, and the chlorobenzene was coated on the film between 14 and 16 s during the second spinning step as an antisolvent. After the spin coating, the precursor films were annealed on a hotplate at 65 or 120 °C for 10 min. Then bis(trifluoromethylsulfonyl)imidelithium salt (Li‐TFSI, Sigma Aldrich) and 4‐*tert*‐butylpyridine (TBP, Sigma Aldrich) codoped 2,2′,7,7′‐tetrakis(*N*,*N*‐di‐pmethoxyphenylamine)‐9,9′‐spirobifluorene (spiroOMeTAD) solution in chlorobenzene (90 mg mL^−1^) was coated on inorganic perovskite layer at 5000 rpm for 30 s to form the hole transport layer. Finally, a gold film (≈80 nm) was thermally evaporated on spiro‐OMeTAD by using a shadow mask to form a device active area of 9 mm^2^ as the top electrode. The flexible inorganic PSCs were fabricated with a structure of PET/ITO/Nb_2_O_5_/CsPbI_2_Br/spiro‐OMeTAD/Au. The ITO film was deposited on the PET substrates by a magnetron sputtering method. The MgF_2_ was deposited on the back of PET by electron beam evaporation as an antireflection coating. The MgF_2_/PET/ITO flexible substrate has an average transmittance of ≈86% in the spectrum region of 400–800 nm and a sheet resistance of ≈8 Ω sq^−1^. The flexible substrate was cleaned with deionized water and ethanol in an ultrasonic bath. The Nb_2_O_5_ films (60 nm) were deposited on the flexible substrates by an e‐beam evaporation method as the electron transport layer. The CsPbI_2_Br perovskite film, spiro‐OMeTAD layer and Au electrode were prepared with the same procedure as on the rigid substrates.

*Device Characterization*: Top‐view and cross sectional images of samples were analyzed by field‐emission SEM (HITACHI, SU‐8020). XRD spectra were obtained using a D/MAX 2400 diffractometer with Cu Kα radiation (Rigaku). The absorption spectra of DMSO adducts were obtained by a UV/Vis NIR spectrophotometer (PerkinElmer, Lambda 950). The current density–voltage (*J*–*V*) curves of PSCs were collected using a Keithley 2400 series sourceMeter under the illumination of an AM 1.5G at 100 mW cm^−2^ irradiance generated by SAN‐EIELECTRIC XES‐40S2‐CE solar simulator, with the intensity calibrated by a NREL‐traceable KG5 filtered silicon reference cell. Both forward and reverse scans were recorded at a scan rate of 0.03 V s^−1^. The EQE spectra of the PSCs were recorded by a QTest Station 2000ADI system. The sheet resistance of ITO film deposited on PET was measured by an SB118 four‐probe meter.

## Conflict of Interest

The authors declare no conflict of interest.

## Supporting information

Supplementary
