# Supplementary material for: Low Temperature Fabrication for High Performance Flexible CsPbI2Br Perovskite Solar Cells
Source: Adv Sci (Weinh). 2018 Sep 15;5(11):1801117. doi: 10.1002/advs.201801117 (PMC6247060; doi:10.1002/advs.201801117)
Supplement: Supplementary file 1 — Supplementary [file ADVS-5-1801117-s001.pdf]

Copyright WILEY-VCH Verlag GmbH & Co. KGaA, 69469 Weinheim, Germany, 2018.

ADVANCED SCIENCE Open Access

Supporting Information

for *Adv. Sci.,* DOI: 10.1002/advs.201801117

Low Temperature Fabrication for High Performance Flexible $CsPbI_2Br$ Perovskite Solar Cells

Hong Jiang, Jiangshan Feng, Huan Zhao, Guijun Li, Guannan Yin, Yu Han, Feng Yan, Zhike Liu, and Shengzhong (Frank) Liu**

Supporting information

Low Temperature Fabrication for High Performance Flexible $CsPbI_2Br$ Perovskite Solar Cells

Hong Jiang, Jiangshan Feng, Huan Zhao, Guijun Li, Guannan Yin, Yu Han, Feng Yan, Zhike Liu, and Shengzhong (Frank) Liu**

H. Jiang, J. Feng, H. Zhao, G. Yin, Y. Han, Prof. Z. Liu, Prof. S. (F.) Liu

Key Laboratory of Applied Surface and Colloid Chemistry, Ministry of Education; Shaanxi Key Laboratory for Advanced Energy Devices; Shaanxi Engineering Lab for Advanced Energy Technology, School of Materials Science and Engineering, Shaanxi Normal University, Xi'an 710119, China.

E-mail: zhike2015@snnu.edu.cn, szliu@dicp.ac.cn

G. Li, Prof. F. Yan

Department of Applied Physics, The Hong Kong Polytechnic University, Hung Hom, Kowloon, Hong Kong.

Prof. S. (F.) Liu

Dalian National Laboratory for Clean Energy; iChEM, Dalian Institute of Chemical Physics, Chinese Academy of Sciences, Dalian, 116023, China.

E-mail: szliu@dicp.ac.cn

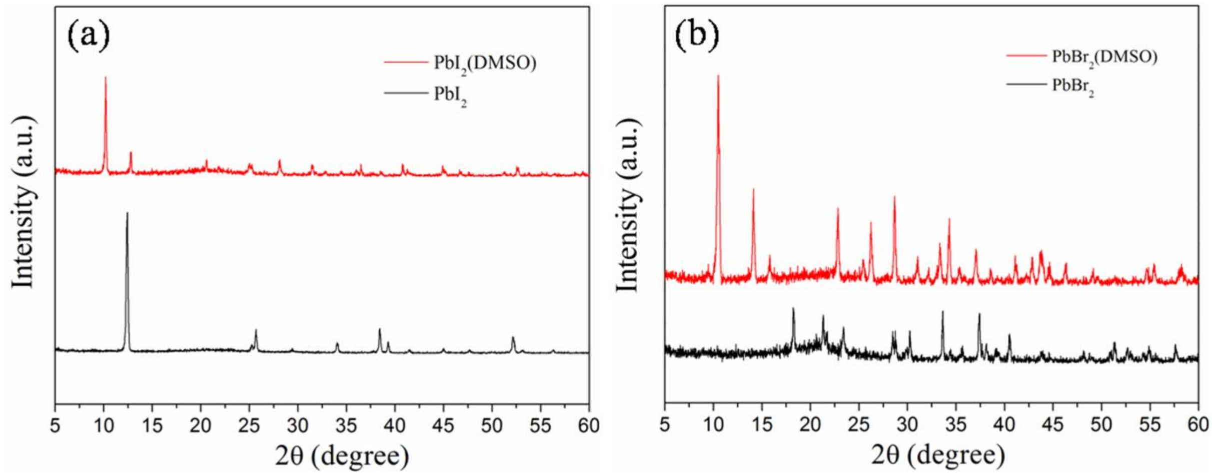

Figure S1. XRD patterns of **(**a) PbI_2 and PbI_2(DMSO) powders. (b) XRD patterns of $PbBr_2$ and $PbBr_2$(DMSO) powders.

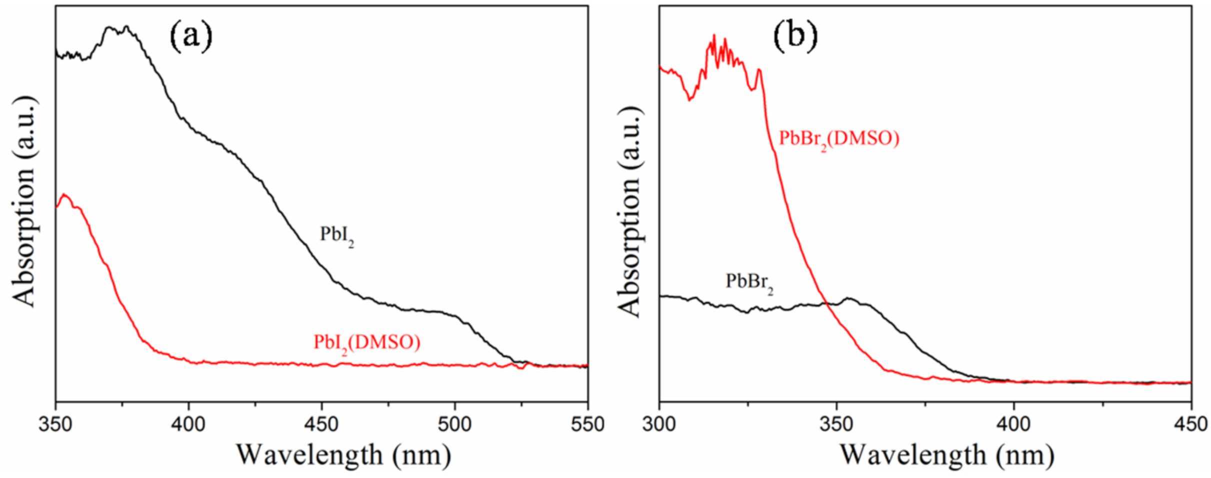

Figure S2. (a) UV-vis absorption spectra of PbI_2 and PbI_2(DMSO) powders. (b) UV-vis absorption spectra of $PbBr_2$ and $PbBr_2$(DMSO) powders.

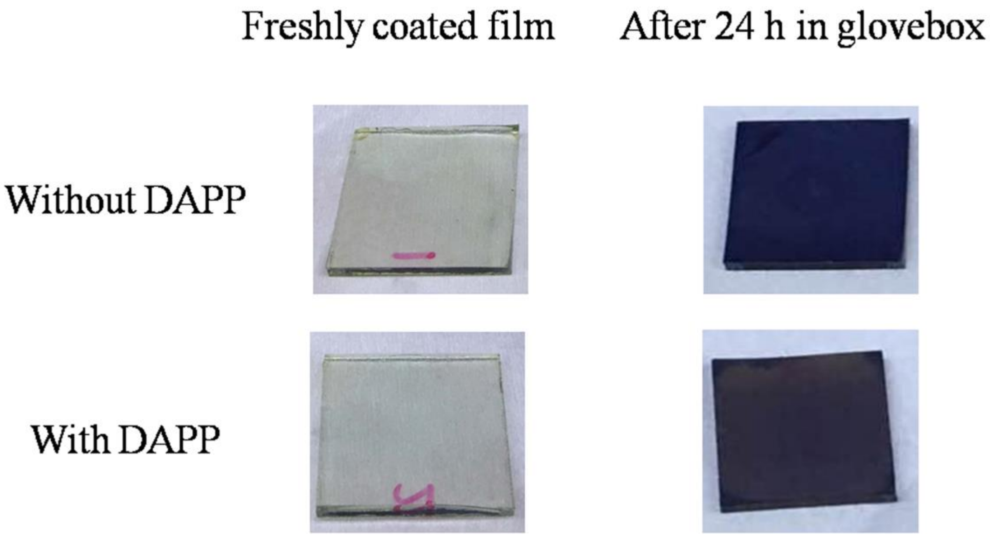

Figure S3. Optical images of $CsPbI_2Br$ films without and with DAPP before and after storing in glovebox for 24 h.

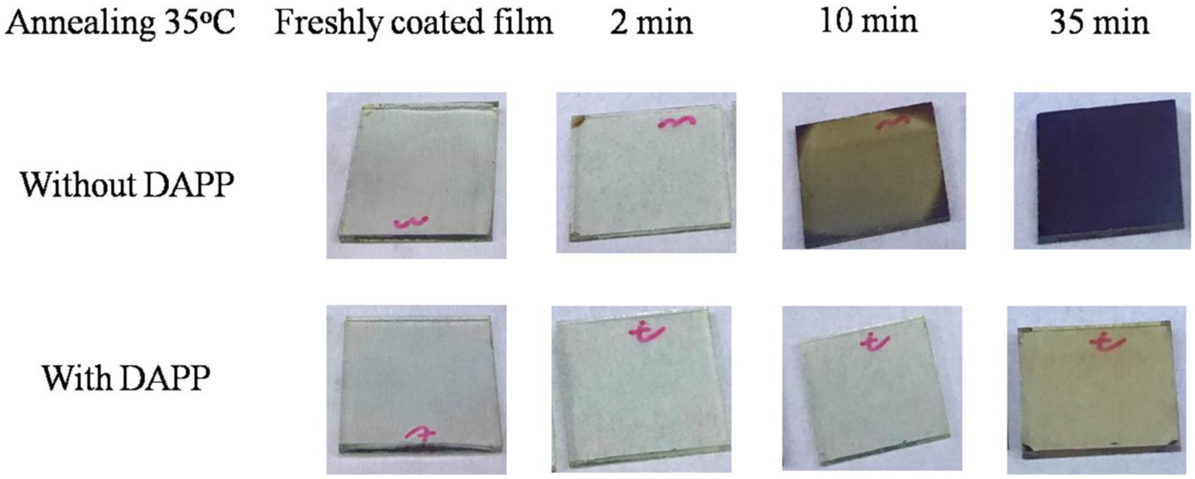

Figure S4. Optical images of $CsPbI_2Br$ films without and with DAPP before and after annealing at 35 ^oC for different times (0/2/10/35 min).

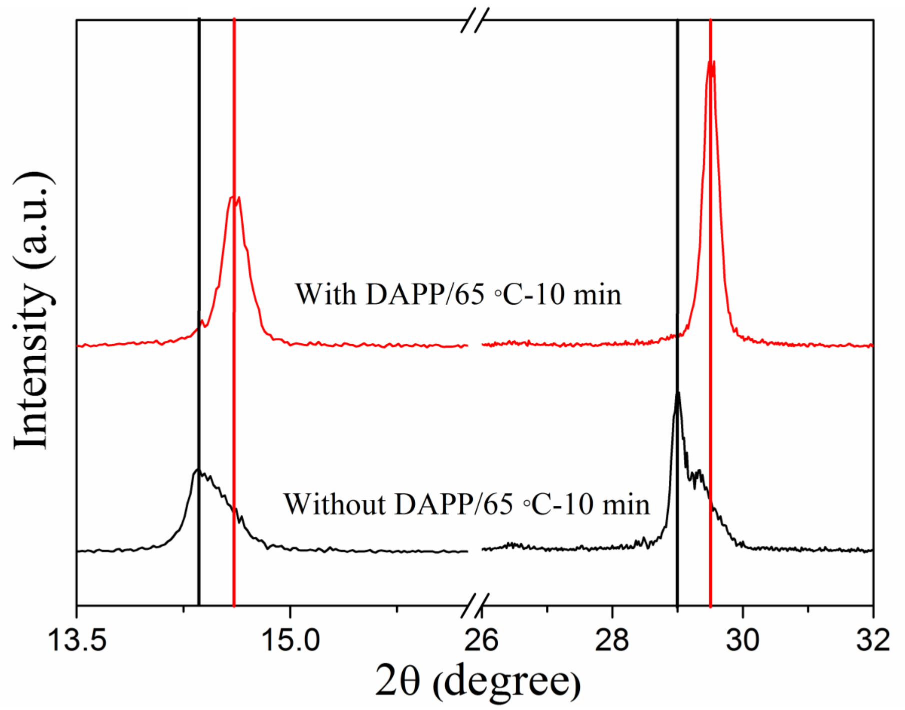

Figure S5. The enlarged XRD patterns of $CsPbI_2Br$ films with and without DAPP annealed at 65 °C for 10 min.

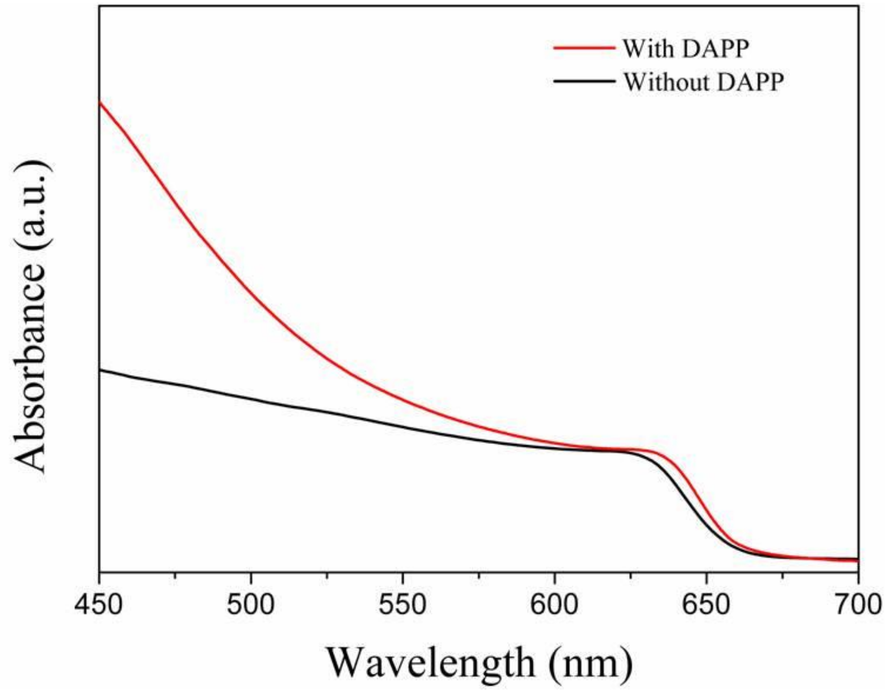

Figure S6. UV-vis absorption spectra of the $CsPbI_2Br$ films prepared without and with DAPP.

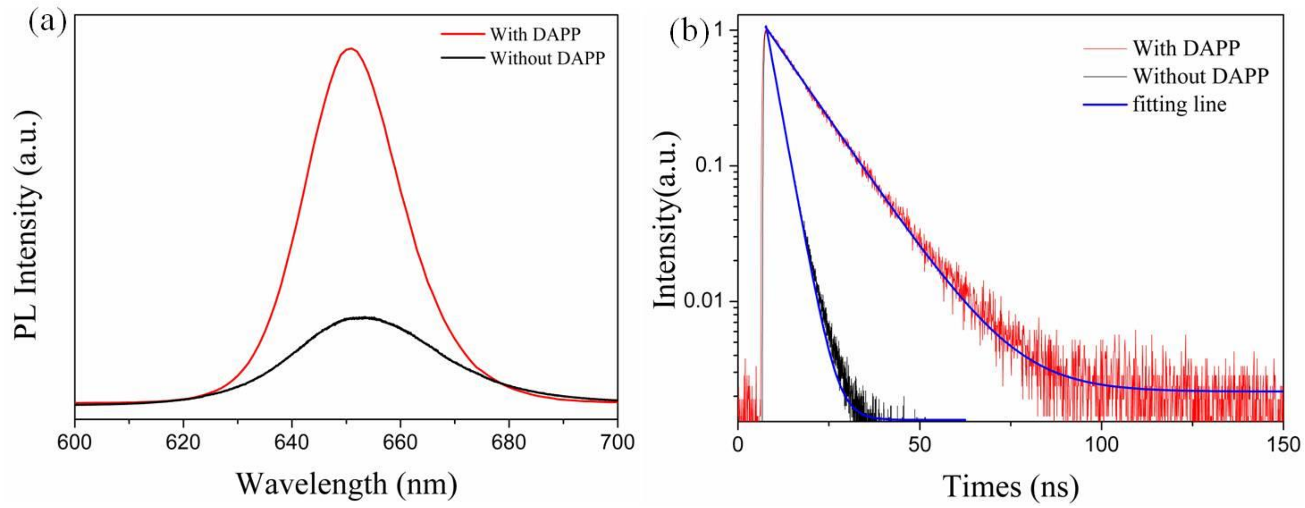

Figure S7. (a) Steady-state PL and (b) time-resolved PL spectra of $CsPbI_2Br$ films prepared without and with DAPP.

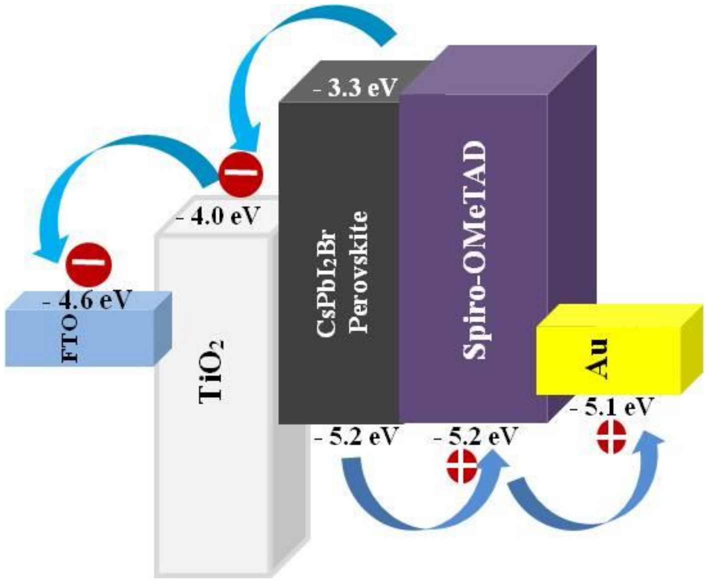

Figure S8. Energy diagram of the rigid $CsPbI_2Br$ PSCs.

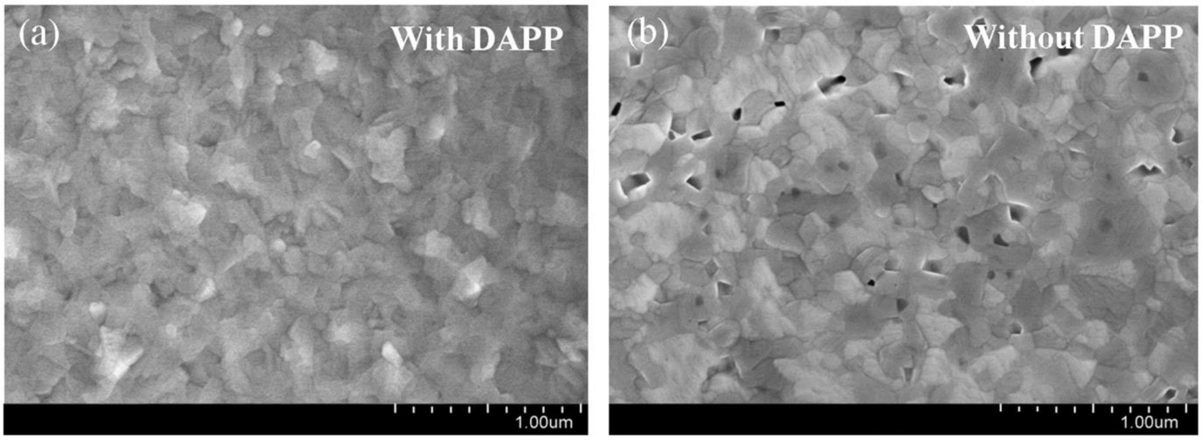

Figure S9. Scanning electron microscopy (SEM) images of the $CsPbI_2Br$ perovskite film (a) with and (b) without DAPP coated on FTO/TiO_2 substrates and annealed at 120 ^{o}C for 10 min.

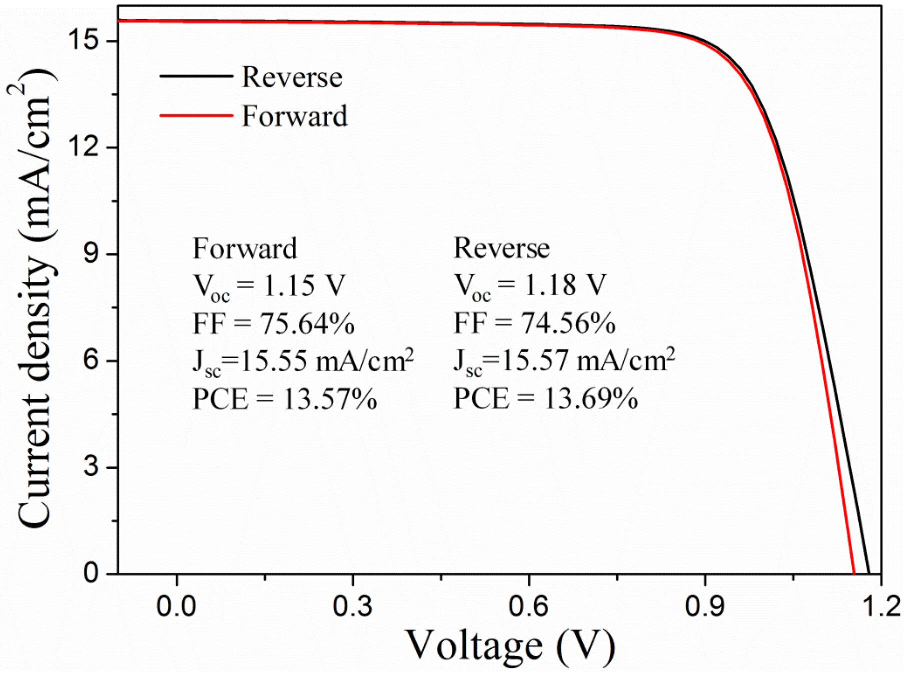

Figure S10. The reverse and forward *J-V* curves of champion $CsPbI_2Br$ PSC with DAPP.

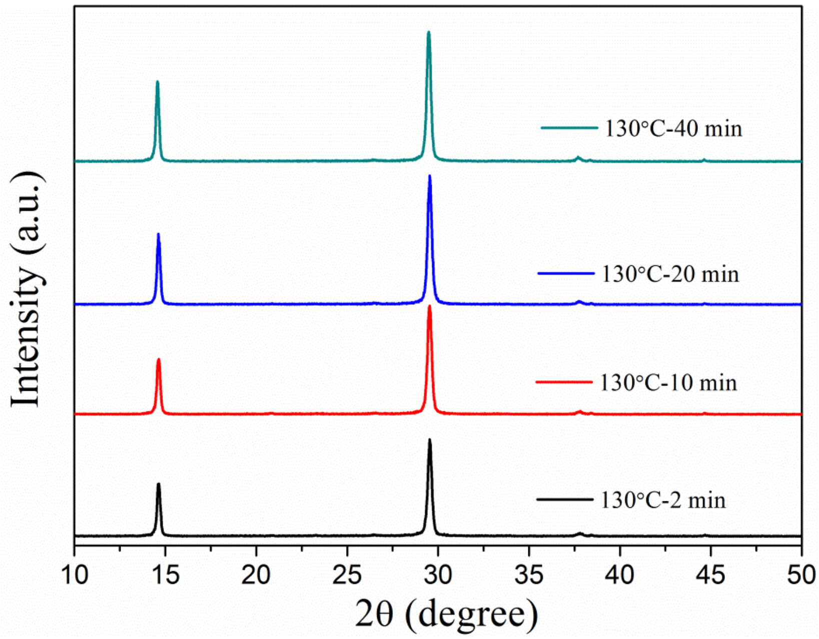

Figure S11. XRD patterns of the $CsPbI_2Br$ perovskite films anneal at 130 ^{o}C for different times (2/10/20/30/40 min).

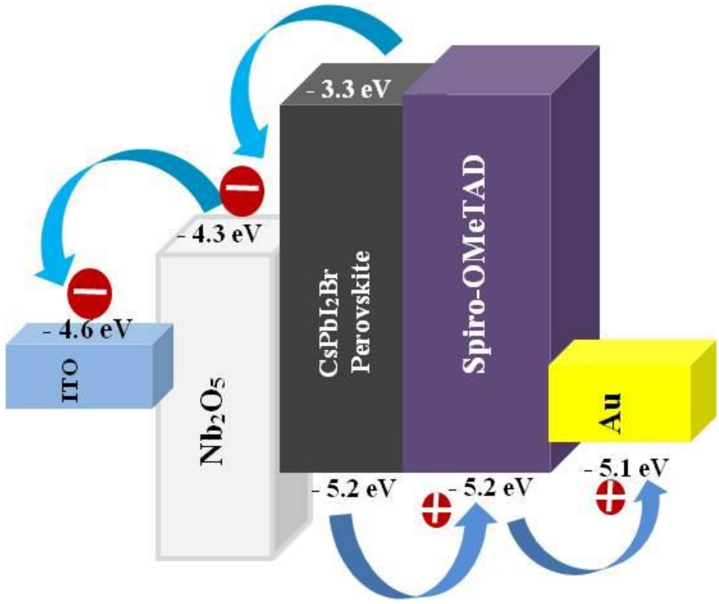

Figure S12. Energy diagram of the flexible $CsPbI_2Br$ PSCs.

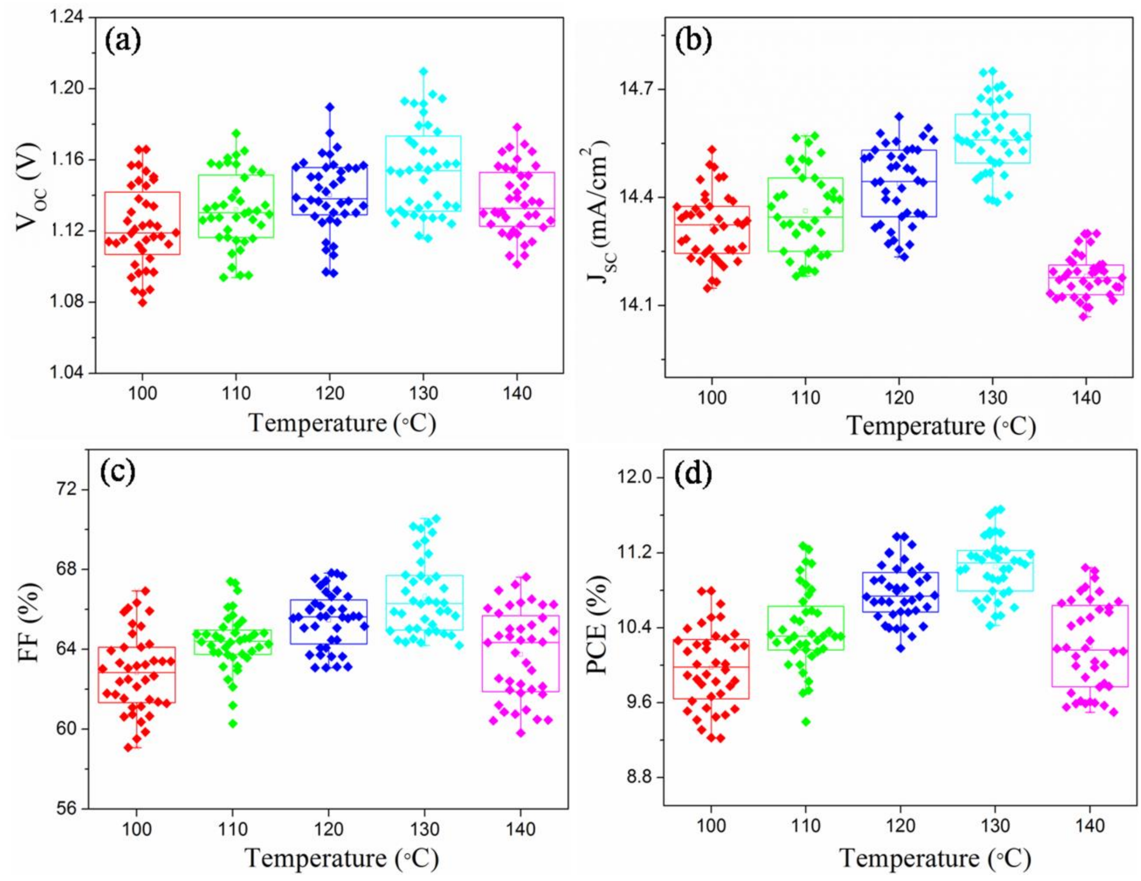

Figure S13. Box charts of (a) open-circuit voltage (V_{OC}), (b) short circuit current (J_{SC}), (c) FF and (d) PCE of $CsPbI_2Br$ PSCs annealed at different temperatures (100/110/120/130/140 ^{o}C).

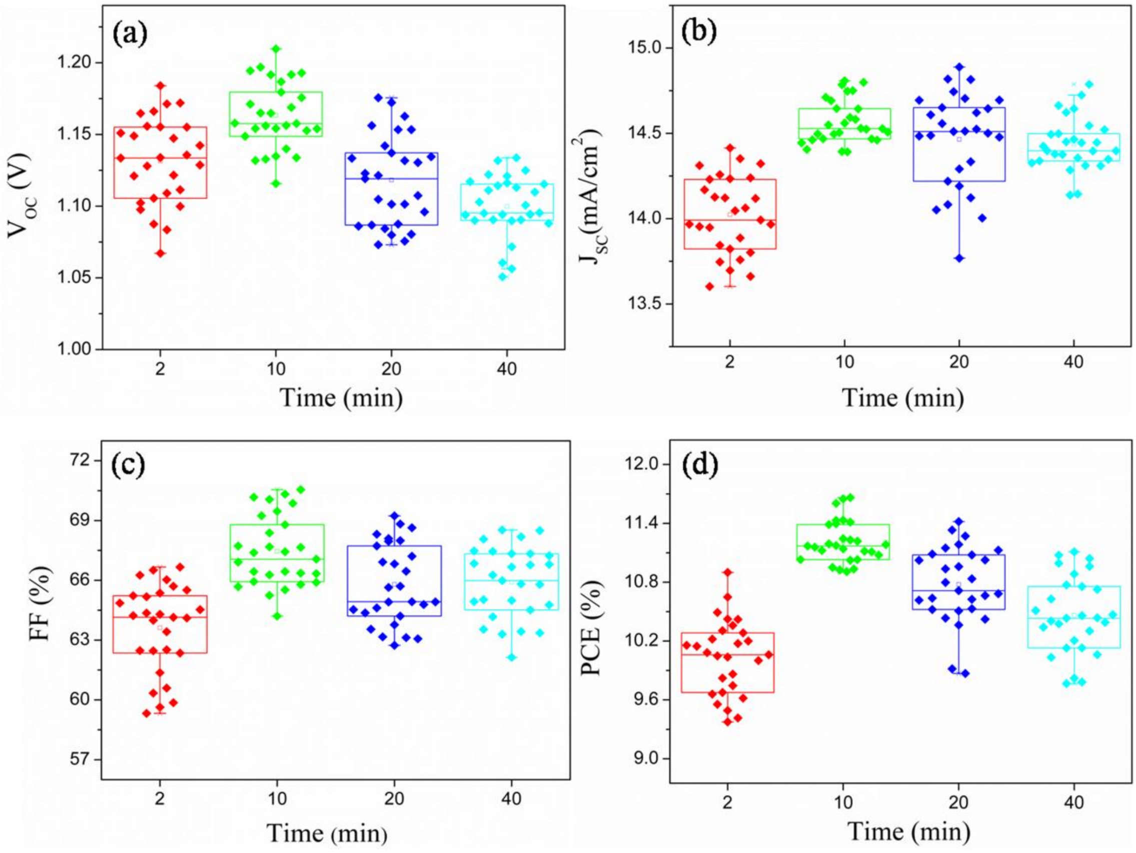

Figure S14. Box charts of (a) V_{OC}, (b) J_{SC}, (c) FF and (d) PCE of $CsPbI_2Br$ PSCs annealed at 130 ^oC for different times (2/10/20/30/40 min).

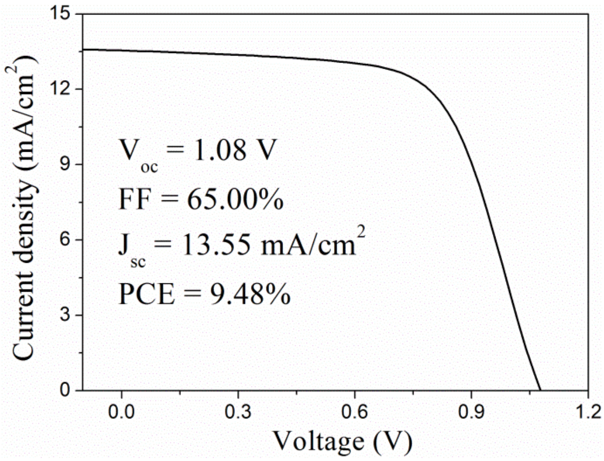

Figure S15. (a) *J*-*V* curve of champion flexible $CsPbI_2Br$ PSC without DAPP under 1 sun (AM 1.5G illumination).

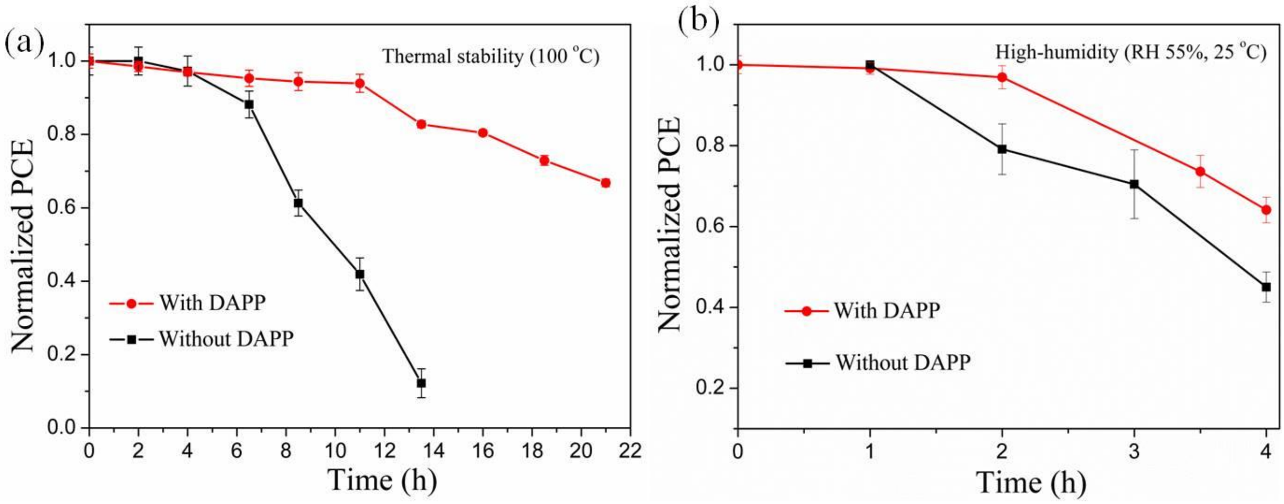

Figure S16. The stability of $CsPbI_2Br$ PSCs with and without DAPP (a) under thermal annealing at 100 °C in the glove box for various durations (b) stored in air with a high- humidity of ~55%.

Tables:

Table S1. Comparison of the performance parameters of the $CsPbI_2Br$ PSCs with DAPP annealed at 130 ^{o}C for different times.

Condition	V_{OC} (V)	J_{SC} (mA/cm^2)	FF (%)	PCE (%)
130℃-2 min	1.17	14.23	65.36	10.90
130℃-10 min	1.19	14.61	67.25	11.73
130℃-20 min	1.12	14.82	68.64	11.42
130℃-40 min	1.13	14.40	68.18	11.11

Table S2. Fitting parameters of the TRPL spectroscopy of $CsPbI_2Br$ films prepared with and without DAPP.

Samples	τ_{ave} (ns)	τ_1 (ns)	τ_2 (ns)	% of τ_1	% of τ_2
Without DAPP	3.412	6.444	2.763	4.69	95.31
With DAPP	11.573	16.095	9.709	19.92	80.08
